# From Waste to Resource: Performance of Black Soldier Fly Larvae Reared on Restaurant Food Waste at an Industrial Scale

**DOI:** 10.3390/insects17040396

**Published:** 2026-04-05

**Authors:** Joana Oliveira, Carolina Ligeiro, Rafaela Fantatto, Clarice Silva e Souza, Maria Ana Machado, Leonardo Gaston Guilgur, Alexandre Trindade, Ricardo Assunção, Daniel Murta

**Affiliations:** 1 Egas Moniz Center for Interdisciplinary Research (CiiEM), Egas Moniz School of Health & Science, 2825-165 Almada, Portugal; 2 Ingredient Odyssey SA—EntoGreen, 2005-002 Santarém, Portugal; 3 Organismal Metabolic Physiology, Gulbenkian Institute for Molecular Medicine (GIMM), 2780-156 Lisbon, Portugal; 4 Food and Nutrition Department, National Institute of Health Dr. Ricardo Jorge, 1649-016 Lisbon, Portugal; 5 Thunder Foods SA, 2005-332 Santarém, Portugal

**Keywords:** sustainable agrifood systems, BSFL bioconversion, circular economy, One Health, food waste

## Abstract

Food waste poses significant environmental, economic and public health challenges. The black soldier fly (*Hermetia illucens*) larvae (BSFL) represent a promising solution for organic waste valorisation, converting substrates into protein-rich biomass for animal feed and organic fertiliser. However, the use of food waste as an insect substrate remains prohibited in the European Union due to regulatory and safety concerns. This study evaluated the suitability of heterogeneous food waste for BSFL rearing under industrial conditions by comparing larval performance on a standard Gainesville diet (control) and a blend derived from local restaurant waste (test). The 14-day bioconversion assay assessed bioconversion rate (BCR), feed conversion ratio (FCR), survival rate, average growth rate, and nutritional composition. Compared with the control, the test group showed significantly improved (*p* < 0.001) BCR (18.34% vs. 11.02%), FCR (5.48 vs. 9.09 kg/kg), survival (69.29% vs. 51.30%), and growth (8.38 vs. 6.59 mg/day). Larvae reared on food waste also exhibited significantly higher protein (19.70% vs. 16.80%), fat (13.70% vs. 7.20%), ash (6.97% vs. 3.51%), carbohydrates (7.00% vs. 3.60%), and fibre (5.20% vs. 2.90%). Overall, heterogeneous food waste is a suitable substrate for BSFL, supporting agrifood sustainability; however, future research should focus on standardisation of these substrates.

## Simple Summary

Food waste is a major environmental and economic problem, posing public health risks. The black soldier fly (*Hermetia illucens*) larvae can convert organic waste into valuable products, including protein-rich biomass for animal feed and frass that may be used as fertiliser. However, in the European Union, food waste is not currently permitted as a substrate for insect rearing due to safety and regulatory concerns. This study evaluated whether heterogeneous food waste from local restaurants could effectively support black soldier fly larval growth under industrial conditions. We compared larval performance on food waste with a standard control diet over 14 days, measuring survival rate, average growth rate, feed conversion ratio, and nutritional composition. Larvae reared on food waste showed improved bioconversion efficiency, higher survival and growth rates, and enhanced nutritional profile compared with the control group. Our findings demonstrate that food waste can be an efficient substrate for insect production if the substrates are properly standardised to ensure safety and consistency, supporting more sustainable and circular agrifood systems.

## 1. Introduction

The global population continues to rise and is projected to increase by 1.4 billion people by 2054, according to the United Nations [1]. This demographic growth intensifies pressure on agrifood systems, demanding higher productivity and greater resource use, while persistent systemic inefficiencies continue to generate substantial losses across the food supply chain [2,3,4,5]. Food waste occurs across the entire supply chain, from production to consumption, and imposes considerable economic, social, and environmental costs [3,5]. In the European Union (EU), more than 59 million tonnes of food are wasted each year, at a cost of 132 billion euros [6,7]. This waste threatens the resilience and sustainability of our agrifood systems, leading to food insecurity: 8.5% of the EU’s population is living in hunger [8]. Beyond these economic implications, inadequate management of organic waste contributes substantially to greenhouse gas emissions and may facilitate environmental contamination and pathogen dissemination, posing potential public health risks [9,10,11,12]. These challenges highlight the urgent need for waste management strategies capable not only of reducing environmental burdens but also of recovering value from organic residues [11,12]. In this context, insect-based bioconversion has emerged as a promising circular economy approach. Insects can efficiently convert organic substrates into valuable biomass rich in protein and lipids, while producing frass that can be used as fertiliser. Compared to conventional livestock systems, insect production generally requires fewer resources and is associated with lower greenhouse gas emissions, reinforcing its potential contribution to more sustainable agrifood systems [13,14,15,16]. From an economic perspective, insect production presents a potential advantage over conventional livestock systems, as it can rely on low-cost or negative-cost substrates such as food waste, thereby reducing feed costs, which are one of the main economic drivers in animal production systems [16].

Among insect species, black soldier fly larvae (BSFL), the larval stage of *Hermetia illucens* [17], are widely recognised as the most common and effective insect used for bioconversion of organic matter for the production of feed and fertilisers. Food waste substrate for BSF provides a dual economic benefit by lowering waste management expenses associated with collection, transport, and disposal, while simultaneously generating value-added biomass rich in protein and lipids [18]. This biomass can be incorporated into animal feed and contribute to the economic sustainability of insect-based production systems [19]. From an economic perspective, the viability of BSFL-based systems is strongly dependent on process performance and efficiency, particularly when scaling up to industrial conditions. Key performance indicators such as larval growth rate, survival rate, bioconversion efficiency, and feed conversion ratio directly influence productivity, processing time, and biomass yield, which are critical determinants of cost-efficiency in large-scale production systems [20].

Overall, previous studies have shown that BSFL can be reared on a wide range of substrates, including agricultural by-products, animal manure, and food waste, making this insect an ideal candidate for sustainable, circular economy-based agrifood systems [18,19,21]. In particular, the use of food waste as a rearing substrate has received increasing attention in recent years. A substantial number of studies have investigated different types of food waste, including kitchen waste, market waste, restaurant waste, fast food waste, and mixed pre- and post-consumer residues [22,23,24,25,26,27,28,29,30]. In general, these studies show that BSFL are able to efficiently convert heterogeneous food waste into biomass, generally achieving good feed conversion ratios (FCR), high survival rates, and increased protein content under controlled conditions. Cheng et al. [29] showed that BSFL can efficiently process both pre- and post-consumer food waste, particularly when substrate moisture content is around 70–75%, supporting larval growth and process efficiency. Efficient biomass production has also been reported when using mixed kitchen waste, with protein content reaching approximately 32–41% [27]. The use of mixed substrates, such as combinations of kitchen waste with soybean curd residue, has also been shown to improve larval biomass, lipid accumulation, and overall conversion efficiency [28]. Similarly, food waste derived from catering environments, including cafeteria waste with both cooked and uncooked fractions, has been associated with high protein content in larvae [25]. Studies comparing multiple waste streams indicate that BSFL can be successfully reared on a wide range of food waste types, with restaurant waste, including fast food residues, often supporting higher larval growth, protein levels, and efficient nutrient conversion, resulting in larvae with high protein and lipid content [22,23,24]. Similarly, studies using fresh market food waste show that the combination of substrates such as fish waste, slaughtered chicken waste, and vegetable waste can produce larvae with enhanced nutritional value, high protein content, high survival rates, and efficient bioconversion [30]. Other food waste streams have also been tested, including pizza, burgers, and pasta dishes, which have been shown to support high larval weight, increased survival rate, and efficient feed conversion [26]. Taken together, these studies show that BSFL can adapt to a wide range of food waste substrates and consistently produce protein- and lipid-rich biomass, although performance remains highly dependent on substrate composition and variability.

Despite these promising findings, most of the available evidence is derived from laboratory or pilot-scale studies. A limited number of full-scale investigations have been conducted, including one study reporting a long-term system processing up to 15 tonnes of domestic biodegradable waste per day, primarily focusing on the economic feasibility of BSFL-based systems, while also reporting crude protein contents that were generally comparable, but slightly lower than those observed under laboratory-scale conditions [31]. These examples remain scarce and insufficient to comprehensively characterise system performance under diverse, real-world conditions. Consequently, there is still a critical knowledge gap regarding BSFL performance at industrial scale when processing heterogeneous food waste. We hypothesise that heterogeneous food waste collected from real food service environments can support efficient BSFL growth and bioconversion under industrial conditions, achieving performance comparable to or exceeding that obtained with standardised reference diets, such as the Gainesville diet. To test this hypothesis, the present study evaluated the suitability of restaurant-derived food waste as a rearing substrate for BSFL at industrial scale. Key performance indicators, including growth rate, survival rate, bioconversion efficiency, and feed conversion ratio, were assessed, together with the nutritional composition of both substrates and larvae. By focusing on non-standardised, real-world waste streams collected from multiple restaurants, this study provides novel insights into the scalability and operational feasibility of BSFL-based food waste valorisation systems.

## 2. Materials and Methods

### 2.1. Insect Rearing

This study was conducted using BSFL provided by EntoGreen (Santarém, Portugal). The larvae had been raised under conditions optimised for their development and were six days old at the beginning of the experiment, corresponding to the second instar stage. Prior to this, they had been fed a growth-promoting diet consisting of wheat bran and water, adjusted to a final moisture content of approximately 75%. All experimental work was conducted at EntoGreen R&D unit’s facilities. Throughout the trial, environmental conditions such as temperature and humidity were monitored to avoid the risk of overheating inside the rearing boxes, which could exceed 47 °C and thereby compromise the survival rate of the larvae.

### 2.2. Substrates and Experimental Design

In this experiment, two types of rearing substrates were used. The control group was fed the Gainesville diet, a standardised, nutritionally balanced rearing substrate commonly used for BSFL, typically consisting of approximately 67% water, 17% wheat bran, 6.6% maize flour, and 9.9% alfalfa, which ensures consistent larval growth and performance [32]. For the test group, a mixture was prepared using 1847.5 kg of heterogeneous food waste collected from nine restaurants located in Santarém, Portugal. Food waste included vegetables and fruits, cereals, cooked and raw meat, cooked and raw fish, eggs, eggshells, and bakery products. The collection was carried out with the support of the Municipality of Santarém, within the InsectERA mobilizing agenda, which provided dedicated waste containers to enable selective collection. The residues were transported under refrigerated conditions to the rearing facilities, where they were subsequently ground and used for substrate formulation. To ensure an adequate texture, wheat bran was added to the food waste blend at 12.5% (*w*/*w*). To ensure consistency and comparability between substrates, NKMix software (version 2.8.72) was used for moisture content adjustment. Moisture content was measured prior to the trial, with the control substrate having 67.23% and the test substrate 65.33%. Both groups were provided with an equal quantity of substrate (13.5 kg per unit). One hundred and fourteen test units and one hundred and forty-four control units were inoculated with substrate, with a higher number of control units included to increase the statistical robustness of the baseline condition. Each unit received 20,000 larvae, which were six days old at the beginning of the experiment, corresponding to the second instar stage. Units were conducted in PVC boxes (60 × 40 × 11 cm) created by EntoGreen. Environmental conditions were monitored throughout the trial, with temperatures ranging from 21.76 to 29.21 °C and relative humidity between 41.99 and 57.11%. The bioconversion process lasted fourteen days and was terminated when the first prepupae began to appear. At the end of the experiment, the larvae were separated from the frass through sieving.

### 2.3. Evaluation of Larval Growth Rate, Feed Conversion Ratio, and Bioconversion Efficiency

Throughout the bioconversion period, key parameters were recorded every other day. These included the weight of a 25 larvae sample from random positions on the crate (larvae were returned to their original rearing boxes after weighing), the weight of the rearing boxes, and the internal temperature within each box. Representative measurements were randomly taken from a total of 64 boxes: 32 from the control group and 32 from the test group. This sampling strategy was designed to strike a balance between practical feasibility and the generation of statistically significant results. The data collected over the assay were later used to assess critical performance indicators, including average larval growth, bioconversion rate (BCR), and feed conversion ratio (FCR). The bioconversion rate is defined as the amount of protein and lipids that the BSFL can produce from a certain quantity of substrate. The greater the bioconversion rate, the more effectively BSFL transform organic matter into valuable products [19,33]. The BCR formula applied in this study was the following [34,35]:

$$\mathrm{BCR}\;(\%)\;\;=\;\frac{\mathrm{final}\;\mathrm{weight}\;\mathrm{of}\;\mathrm{larvae}}{\mathrm{initial}\;\mathrm{weight}\;\mathrm{of}\;\mathrm{substrate}}\times 100$$

FCR is defined as the amount of feed (on a dry matter basis) required to produce a unit of BSFL biomass. Lower FCR values indicate higher efficiency [36,37].

$$\mathrm{FCR}=\frac{\mathrm{total}\;\mathrm{feed}\;\mathrm{consumed}}{\mathrm{total}\;\mathrm{BSFL}\;\mathrm{biomass}}$$

The average growth was calculated as follows [38]:

$$\mathrm{Average}\;\mathrm{growth}\;=\frac{\mathrm{final}\;\mathrm{weight}\;\mathrm{of}\;\mathrm{larvae}-\mathrm{initial}\;\mathrm{weight}\;\mathrm{of}\;\mathrm{larvae}}{\mathrm{number}\;\mathrm{of}\;\mathrm{days}}$$

Additionally, the larval survival rate was calculated to assess overall viability throughout the bioconversion period, using the following formula [35]:

$$\mathrm{Survival}\;\mathrm{rate}\;\;=\;\frac{\mathrm{number}\;\mathrm{of}\;\mathrm{larvae}\;\mathrm{at}\;\mathrm{the}\;\mathrm{end}\;\mathrm{of}\;\mathrm{the}\;\mathrm{assay}}{\mathrm{number}\;\mathrm{of}\;\mathrm{larvae}\;\mathrm{at}\;\mathrm{the}\;\mathrm{start}\;\mathrm{of}\;\mathrm{the}\;\mathrm{assay}}\times 100$$

### 2.4. Nutritional Profile of Substrates, Larvae and Meal

Samples of substrates, larvae, and processed meal from both control and test groups were collected to determine their nutritional composition. Substrate samples were obtained on the first day of the assay, prior to larval inoculation, from randomly selected rearing boxes. Larvae were randomly sampled on the final day of the assay after separation from frass, and meal samples were collected after larval processing. For each matrix, six replicates were collected. From these, three were randomly selected for nutritional analysis, and each selected replicate was analysed in triplicate. The analyses were carried out at SGS Multilab (Lisbon, Portugal). The parameters analysed and the methodology employed are stated in Table 1.

The resulting nutritional composition of the substrates is presented in Table 2.

### 2.5. Statistical Analysis

All statistical analyses were performed using IBM SPSS Statistics (version 29.0.2.0, IBM Corp., Armonk, NY, USA). An independent-samples t-test was used to evaluate differences in BCR, FCR, average growth, and temperature between control and test substrates. Statistical significance was set at *p* < 0.05.

Nutritional parameters of larvae reared on the control substrate were compared with those reared on the test substrate. Data were obtained from three independent replicates per treatment. Differences between groups for each nutritional parameter were assessed using the Mann–Whitney *U* test. For samples with results below the limit of quantification (LOQ), a value of LOQ/2 was assigned for statistical analysis.

## 3. Results

### 3.1. Larval Performance Indicators

A significant difference in BCR was observed between groups (t(38.74) = −30.30, *p* < 0.001), with higher values recorded in the test group than in the control. The FCR also differed significantly between treatments (t(62) = 37.35, *p* < 0.001), indicating improved feed efficiency in larvae reared on the test substrate. Similarly, the average growth rate was significantly higher in the test group than in the control (t(53.85) = −6.20, *p* < 0.001). Furthermore, the survival rate was significantly higher in the test group than in the control (t(52.89) = −9.93, *p* < 0.001). Mean values and standard deviations for all these variables are summarised in Table 3.

**Figure 1:**
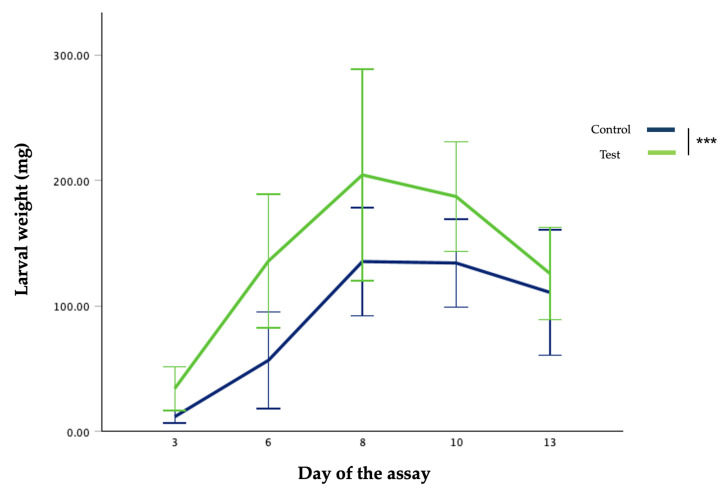
Larvae’s individual weight per day of the assay. *** = *p* < 0.001.

**Figure 2:**
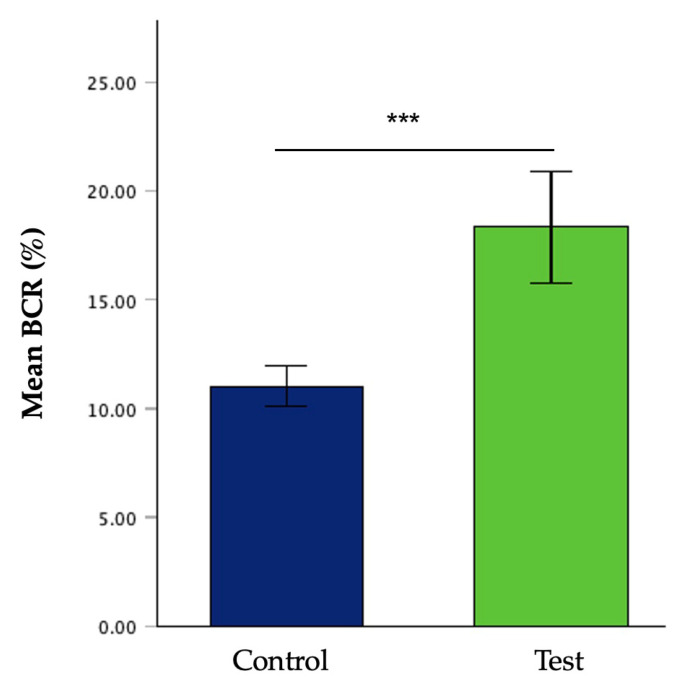
Bioconversion rate mean (%) and standard deviation in both larvae groups. *** = *p* < 0.001.

Figure 1 and Figure 2 illustrate the evolution of larvae’s weight per day and bioconversion rate, respectively.

**Figure 3:**
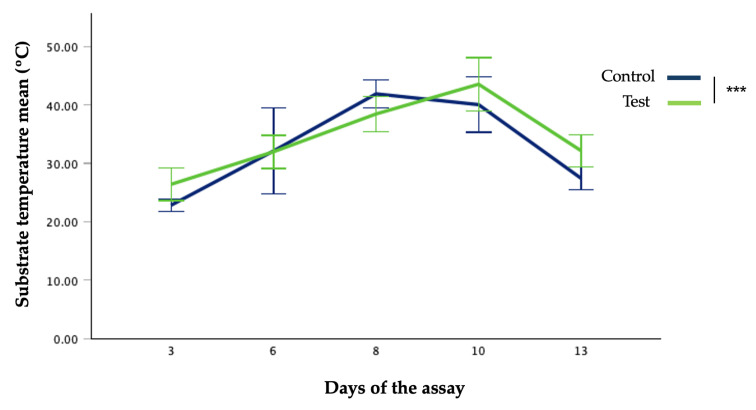
Temperature of substrates from both groups throughout the days of the assay. *** = *p* < 0.001.

As temperatures above 47 °C within the substrate can lead to suboptimal growth, reduced yields, or even larval mortality, effective temperature management is essential for successful BSFL farming [39,40]. Given that microbial activity and larval metabolism can generate significant heat within the substrate, temperature was continuously monitored to assess potential thermal accumulation inside the rearing boxes. This monitoring ensured that conditions remained within a biologically safe range in light of possible thermal stress. Table 4 illustrates the evolution of substrate temperature in the rearing boxes (mean and standard deviation) and Figure 3 shows the evolution of temperature throughout the assay days. Significant differences in temperature between groups were observed on day 3 (*t*(40.559) = −13.569, *p* < 0.001), day 8 (*t*(59.075) = 10.135, *p* < 0.001), and day 10 (*t*(61.906) = −6.033, *p* < 0.001). In contrast, no significant differences were found on day 6 (*t*(40.033) = 0.191, *p* = 0.849) and day 13 (*t*(31.636) = −0.936, *p* = 0.356).

Temperatures in the rearing units ranged from 21 °C to 45 °C in the control group, and from 23 °C to 47 °C in the test group. Exposure to temperatures approaching or exceeding the critical temperature limit (~47 °C) has been shown to impair larval growth and, in some cases, to cause mortality [39].

### 3.2. Nutritional Profile

In addition to differences in growth performance, the substrate type also influenced the nutritional composition of the larvae. Larvae reared on the test substrate had significantly higher contents of carbohydrates, protein, fat, ash, monounsaturated fatty acids, sodium, and dietary fibre than those reared on the control substrate (U = 0.00; *p* < 0.05). In contrast, no significant differences were observed for saturated, polyunsaturated, or trans fatty acids, nor for total sugars. Table 5 presents the median values and corresponding *p*-values and U-values ((Mann–Whitney-U test) for larvae reared on the control and test substrates.

## 4. Discussion

This study aimed to evaluate the suitability of food waste as a substrate for BSFL rearing under industrial conditions, by assessing larval performance (average growth rate, BCR, survival rate and FCR) and its nutritional composition. The test group showed significantly higher growth rates and bioconversion efficiency, along with improved feed conversion ratios, compared with the control. These results suggest that the test conditions enhanced larval development and resource utilisation. These improvements may be attributed to the fact that food waste provides a rich and diverse mix of nutrients, such as proteins, carbohydrates, and fats, that support rapid larval growth and high survival rates [30,39,41], a pattern also observed in this study. However, the intrinsic heterogeneity of food waste represents an important source of experimental variability. Although the substrate used in this study was mechanically processed prior to use, resulting in a homogenised mixture with a particle size of approximately 5–10 mm, the variability in its original composition remains a relevant factor. Previous research has demonstrated that substrate particle size is a key factor influencing BSFL performance, with sizes ranging from 4 to 10 mm enhancing waste reduction (up to 35%) and larval biomass production (up to 38%) compared to finer substrates (<2 mm), while also promoting more efficient carbon and nitrogen bioconversion [42].

Fluctuations in nutrient composition within these substrates may condition larval performance and chemical profiles, which can limit standardisation and comparability across studies. Despite this variability within substrates, BSFL are highly adaptable and polyphagous, meaning they can efficiently digest a wide range of organic materials, aided by their robust digestive system and beneficial gut microbiota, which help break down complex food waste and enhance nutrient absorption [41,42,43]. Similar trends in FCR were reported by Franco et al. (2025), who used meat and fish-based former foodstuffs as a substrate for BSFL rearing [26]. Taufek et al. (2023) also observed comparable results in both growth rate and FCR when rearing BSFL on food waste collected from a fresh market [30], while X. Li et al. (2022) achieved similar bioconversion rates using a mixture of soybean curd and kitchen waste as a substrate [28]. In addition to enhanced growth performance, the nutritional composition of the larvae was also influenced by the substrate type. Larvae reared on the test substrate showed significantly higher levels of protein, fat, ash, and carbohydrates, reflecting the richer nutrient profile of the food waste used. Comparable outcomes have been reported in previous studies, where nutrient-dense substrates led to improved larval macronutrient composition [24,26,28,30]. These findings indicate that substrate composition directly shapes the biochemical profile of BSFL, which is crucial for optimising their use in feed and food applications.

It is important to note that many previous studies have relied on more uniform or single-source substrates. In contrast, the present study used a heterogeneous mixture combining multiple food waste streams collected from restaurants, encompassing a wide range of organic materials as described in Section 2. Importantly, the scale and heterogeneity of the substrate used in this study strengthen the external validity of the findings. Unlike most previous research, which has largely relied on controlled laboratory conditions and standardised or homogeneous substrates [26,28,29,30,43,44], the present work evaluated BSFL performance using a heterogeneous mixture of food waste collected from nine commercial restaurants and processed at industrial scale with about two tonnes of input material. The consistent larval performance observed, despite substrate variability, demonstrates that efficient bioconversion can be achieved under realistic operational conditions, thereby supporting the practical feasibility and scalability of BSFL-based waste valorisation systems. These findings are in line with previous full-scale evidence reported in the literature [31], reinforcing the feasibility of BSFL-based systems under industrial conditions. However, in contrast to that study, which applied a microbial additive followed by a fermentation process to optimise the substrate, the present work specifically focused on untreated restaurant-derived food waste, without any additives or pre-processing steps, while still yielding comparable protein and fat content in the produced larvae. This distinction is particularly relevant, as it allows the assessment of BSFL performance under conditions that more closely reflect real-world waste streams, where such optimisation strategies are not always feasible. Interestingly, although overall larval performance improved under treatment conditions, temperatures in the rearing units occasionally approached the upper thermal threshold for BSFL (~47 °C), which is known to impair larval survival and growth. This thermal increase likely reflects enhanced metabolic heat production driven by larval feeding and microbial substrate degradation, combined with high larval density and aggregation, which restrict heat dissipation and promote localised thermal hotspots [45]. From an industrial standpoint, these findings highlight the need for careful control of larval density, substrate load, and aeration to prevent thermal “runaway” events, in which self-amplifying heat production compromises system stability, productivity, and welfare outcomes. Continuous temperature monitoring and adaptive process management are therefore crucial for safe and scalable BSFL production.

Overall, our results support the viability of using food waste at an industrial scale as a substrate, in insect farming, offering a sustainable alternative for organic waste valorisation. However, important challenges remain. The inherent variability of food waste complicates substrate standardisation, which may require further optimisation to ensure large-scale implementation. Finally, concerns about microbial and chemical safety, including the potential presence of pathogenic microorganisms and the accumulation of undesirable chemical contaminants, may pose risks to animal and human health, particularly since food waste may be inherently contaminated, and therefore, require further investigation.

## Figures and Tables

**Table 1:** Methodology employed on the nutritional profile analysis.

Parameter	Method
Total carbohydrates (%)	Indirect (calculation)
Energy (kcal/100 g)	Indirect (calculation)
Fat content (%)	Solvent extraction
Ash content (%)	Thermogravimetry
Moisture content (%)	Thermogravimetry
Protein (%)	Combustion method
Saturated fatty acids (g/100 g)	Gas Chromatography-Flame Ionization Detector (GC-FID)
Monounsaturated fatty acids (g/100 g)	Gas Chromatography-Flame Ionization Detector (GC-FID)
Polyunsaturated fatty acids (g/100 g)	Gas Chromatography-Flame Ionization Detector (GC-FID)
Trans fatty acids (g/100 g)	Gas Chromatography-Flame Ionization Detector (GC-FID)
Sodium (g/100 g)	Flame Atomic Absorption Spectrometry (FAAS)
Alimentary fiber (%)	Total Dietary Fiber Assay Kit. 200 assays. Megazyme
Total sugars (g/100 g)	High-Performance Liquid Chromatography with Refractive Index Detection (HPLC-RID)

**Table 2:** Nutritional composition of control substrate and test substrate (mean and standard deviation).

Nutritional Profile	Control Substrate	Test Substrate
Total carbohydrates (%)	23.67 ± 0.58	18.66 ± 1.53
Energy (kcal/100 g)	113.00 ± 5.77	150.00 ± 0.00
Fat content (%)	1.30 ± 0.0	7.00 ± 0.17
Ash content (%)	1.83 ± 0.08	3.52 ± 0.48
Moisture content (%)	68.50 ± 0.96	63.76 ± 0.35
Protein (%)	4.87 ± 0.21	7.17 ± 1.12
Saturated fatty acids (g/100 g)	0.32 ± 0.04	2.11 ± 0.19
Monounsaturated fatty acids (g/100 g)	0.34 ± 0.11	3.29 ± 0.06
Polyunsaturated fatty acids (g/100 g)	0.72 ± 0.13	1.57 ± 0.02
Trans fatty acids (g/100 g)	<loq ^1^	<loq ^1^
Sodium (g/100 g)	0.02 ± 0.00	0.21 ± 0.01
Alimentary fibre (%)	6.13 ± 0.15	6.20 ± 0.53
Total sugars (g/100 g)	2.30 ± 0.10	4.13 ± 0.21

^1^ Loq—limit of quantification for trans fatty acids is 0.10 g/100 g.

**Table 3:** Mean values and standard deviations for BCR, FCR, average growth rate, and survival rate in control and test groups.

Parameter	Group	Mean with Standard Deviation
**BCR (%)**	Control	11.02 ± 0.46
	Test	18.34 ± 1.29
**FCR (kg/kg)**	Control	9.09 ± 0.38
	Test	5.48 ± 0.39
**Average growth rate (mg/day)**	Control	6.59 ± 0.90
	Test	8.38 ± 1.36
**Survival rate (%)**	Control	51.30 ± 5.51
	Test	69.29 ± 8.58

**Table 4:** Temperature evolution in the rearing boxes during the experimental period for both control and test conditions.

Bioconversion Assay Day	Temperature of Substrate (°C)	
	Control	Test
3	22.8 ± 0.6	26.4 ± 1.4
6	32.1 ± 3.7	32.0 ± 1.4
8	41.9 ± 1.2	38.4 ± 1.5
10	40.0 ± 2.4	43.5 ± 2.3
13	29.9 ± 13.7	32.1 ± 1.4

**Table 5:** Nutritional profile median for larvae, *p*-values and U-values.

Median	Control Larvae	Test Larvae	*p*-Value	U
Total carbohydrates (%)	3.60	7.00	<0.05	0.00
Energy (kcal/100 g)	140.00	220.00	0.025	0.00
Fat content (%)	7.20	13.70	<0.05	0.00
Ash content (%)	3.51	6.97	<0.05	0.00
Moisture content (%)	69.10	52.10	<0.05	0.00
Protein (%)	16.80	19.70	0.046	0.00
Saturated fatty acids (g/100 g)	3.46	5.69	0.127	1.00
Monounsaturated fatty acids (g/100 g)	1.24	5.12	<0.05	0.00
Polyunsaturated fatty acids (g/100 g)	2.38	2.49	0.658	3.50
Trans fatty acids (g/100 g)	0.05	0.05	1.00	4.50
Sodium (g/100 g)	0.04	0.15	0.037	0.00
Alimentary fiber (%)	2.90	5.20	0.046	0.00
Total sugars (g/100 g)	0.03	0.03	1.00	4.50

## Data Availability

Data is available upon request.

## Abbreviations

The following abbreviations are used in this manuscript:

BCR	Bioconversion rate
BSFL	Black soldier fly larvae
FCR	Feed conversion ratio
LOQ	Limit of quantification
USD	United States Dollar
